# Supplementary material for: Cosmos 1.0: a multidimensional map of the emerging technology frontier
Source: Sci Data. 2025 Nov 19;12:1837. doi: 10.1038/s41597-025-06125-y (PMC12630975; doi:10.1038/s41597-025-06125-y)
Supplement: Supplementary file 1 — Supplemental Information [file 41597_2025_6125_MOESM1_ESM.docx]

**Supplemental Information**

1. **Supplementary Methods**

**Instance label**: academic discipline, academic major, access control, advanced driver-assistance systems, advertising, aircraft, aircraft model, algorithm, allotrope of silicon, animation technique, application, applications of artificial intelligence, applied science, art genre, artificial intelligence model, artificial satellite, audio effect, automated rapid transit, automation, autonomous car, battery chemistry type, Berkeley Open Infrastructure for Network Computing projects, biological process, branch of biology, branch of chemistry, branch of computer science, branch of physics, branch of science, business, buzzword, carbon neutrality, cellular network, certification, chatbot, chemical element, cinematic technique, class of fictional entities, climate change mitigation, cloud computing, communication protocol, communication technology, computer graphics term, computer network protocol, computer program, computer simulation, computing, computing platform, concept, copy protection, design, digital rights management, distributed computing, distributed revision control system, driver of Industry 4.0, drug delivery, educational software, educational technology, electrical element, email filtering, energy conservation technology, energy industry, error correction code, expert system, fictional technology, fictional uploaded consciousness, field of study, finance, forward error correction, free and open-source software, free software, genetic algorithm, grid computing, group or class of chemical substances, group or class of proteins, GNU package, hash function, history of technology, hypothetical technology, image processing, individual transportation, industry, information system, information technology, informed search algorithm, integrated development environment, interdisciplinary science, interface standard, invention, knowledge market, learning management system, light rail, linear code, list of manufacturing processes, machine learning, macroscopic quantum phenomena, marine propulsion, media, medical device type, medical diagnosis, medical imaging, medical journal, medical model, medical speciality, medical test type, metaheuristic, method, mobile app, mobile phone generation, mobile phone network standard, mobile telecommunication technology, mode of transport, navigation system, network, noise reduction, non-classical state of matter, nuclear reactor generation, nuclear technology, online course, online service, open-source software, parody generator, patent classification, pathfinding algorithm, people mover, periodical, peripheral, photographic technique, physical media format, physical technological component, process, product, production process, programming language, programming paradigm, project, property, protocol stack, public-domain software, push technology, question-and-answer site, radar, radio program, reasoning, recording medium, research project, robot, robotics, scholarly article, science, science fiction theme, scientific journal, sensor, sequencing, server software, service, social networking service, software, software as a service, software feature, software library, space instrument, spaced repetition software, spaceflight, speciality, startup company, subject heading, superpower, symbol, system, Tactical communications system, technical sciences, technical standard, technical term, technique, technology, thermal weapon sight, thermographic camera, thought experiment, trademark, train and rail category, Transport system for persons, type of computer memory or storage, type of manufactured good, type of quantum particle, type of security, type of sport, type of technology, vaccine type, vehicle model, virtual reality, visual effects, volunteer computing, weapon functional class, web portal, web service, website, Wikimedia disambiguation page.
